# Homing and Long-Term Engraftment of Long- and Short-Term Renewal Hematopoietic Stem Cells

**DOI:** 10.1371/journal.pone.0031300

**Published:** 2012-02-09

**Authors:** Liansheng Liu, Elaine F. Papa, Mark S. Dooner, Jason T. Machan, Kevin W. Johnson, Laura R. Goldberg, Peter J. Quesenberry, Gerald A. Colvin

**Affiliations:** 1 Department of Medicine, Division of Hematology/Oncology, Rhode Island Hospital, Providence, Rhode Island, United States of America; 2 Department of Biostatistics, Rhode Island Hospital, Providence, Rhode Island, United States of America; 3 Departments of Orthopaedics and Surgery, The Warren Alpert Medical School of Brown University, Providence, Rhode Island, United States of America; French Blood Institute, France

## Abstract

Long-term hematopoietic stem cells (LT-HSC) and short-term hematopoietic stem cells (ST-HSC) have been characterized as having markedly different *in vivo* repopulation, but similar *in vitro* growth in liquid culture. These differences could be due to differences in marrow homing. We evaluated this by comparing results when purified ST-HSC and LT-HSC were administered to irradiated mice by three different routes: intravenous, intraperitoneal, and directly into the femur. Purified stem cells derived from B6.SJL mice were competed with marrow cells from C57BL/6J mice into lethally irradiated C57BL/6J mice. Serial transplants into secondary recipients were also carried out. We found no advantage for ST-HSC engraftment when the cells were administered intraperitoneally or directly into femur. However, to our surprise, we found that the purified ST-HSC were not short-term in nature but rather gave long-term multilineage engraftment out to 387 days, albeit at a lower level than the LT-HSC. The ST-HSC also gave secondary engraftment. These observations challenge current models of the stem cell hierarchy and suggest that stem cells are in a continuum of change.

## Introduction

Elegant work, utilizing fluorescently labeled monoclonal antibodies and fluorescence-activated cell sorting (FACS), has progressively characterized the multilineage repopulation potential of different marrow cell populations. This work has formed the basis for a detailed marrow stem/progenitor cell hierarchy [1]–[23] in which the most primitive stem cells differentiate into progressively more mature marrow cells with gains of specific function and loss of proliferative, renewal, and total differentiation potential. In this generally accepted model, the most primitive cell is the long-term hematopoietic stem cell separated on the basis of lineage negative status (Lin^−^) and expression of the surface epitopes c-kit and Sca-1 with either intermediate Thy-1.1 expression or absence of Flk-2 [7]. This cell has long-term repopulation and secondary repopulation potential in lethally irradiated mice. Differentiation of the LT-HSC into ST-HSC, a cell with a repopulation potential not exceeding 8-12 weeks, is then characterized by the gain of Flk-2 expression. Loss of Thy-1.1 expression with full expression of Flk-2 characterizes the next differentiation step to the multipotent progenitor (MPP). Further differentiation and subdivision of these cells is then characterized by additional selective epitope expression. LT-HSC and ST-HSC subsetted by cycle status into stem cells capable of long-term and short-term engraftment showed equivalent proliferative potential in *in vitro* liquid culture stimulated by cytokines [21]. The investigators speculated that the difference between these cells might be based on differences in marrow homing capacity. Accordingly, we initiated studies on whether the route of administration of the marrow, intravenous, intraperitoneally, or intrafemoral would affect the engraftment outcomes of Lin^−^c-kit^+^Sca-1^+^Flk-2^−^ (LT-HSC) or Lin^−^c-kit^+^Sca-1^+^Flk-2^+^ (ST-HSC/MPP) marrow cells. We found that administering ST-HSC/MPP by intrafemoral or intraperitoneal routes did not enhance their engraftment potential, but also observed that the Lin^−^c-kit^+^Sca-1^+^Flk-2^+^ (ST-HSC/MPP) marrow cells gave rise to long-term stable engraftment. This work is presented below.

## Results

**Figure 1:**
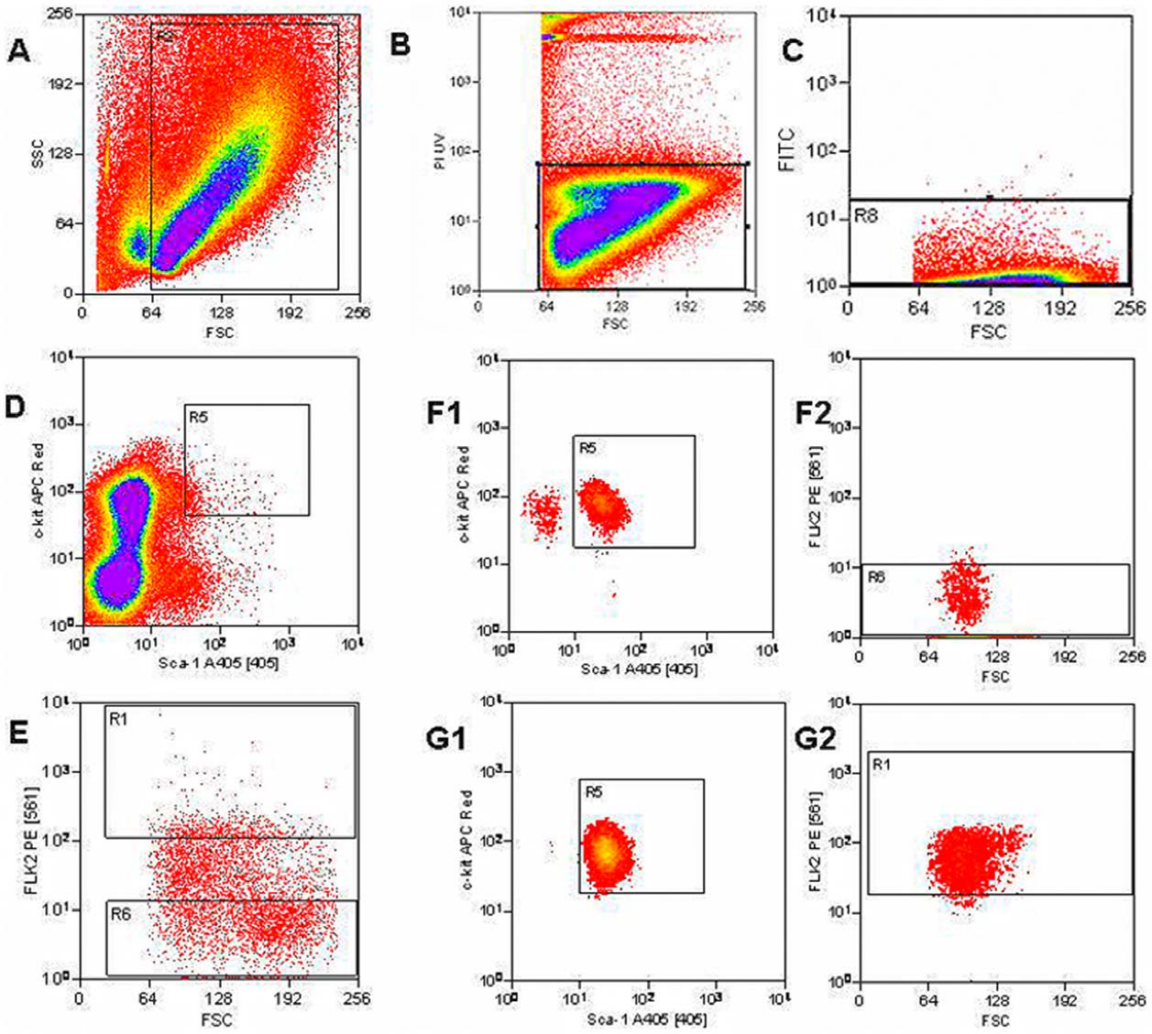
Sorting Scheme. Fluorescence-activated cell sorting of lineage negative (FITC ^−^) leukocytes to isolate long term- and short term-hematopoietic stem cell groups. A) Forward and side scatter plot isolates cells. B) Forward scatter and PI plot captures living cells (PI^−^). C) Forward scatter and FITC plot removes remaining lineage^+^ cells (FITC^+^) and retains lineage^−^ cells (FITC ^−^). D) Sca-1 (Alexa Fluor 405^+^) and c-kit (APC ^+^) positive cells are isolated. E) Forward scatter and Flk-2 plot separates long term cells (Flk-2^−^ PE^−^) from short term cells (Flk-2^+^ PE^+^). Stem cells in the LT-HSC (Flk-2^−^) (F1, F2) and ST-HSC (Flk-2^+^) (G1, G2) groups were resorted to ensure that contaminating cells are not present in either population.

Marrow from B6.SJL donor mice was harvested, lineage depleted, and stained with antibodies to c-kit, Sca-1, and Flk-2 as outlined in Methods (Figure 1). Cells were selected for c-kit^+^, Sca-1^+^, and either Flk-2 negative or Flk-2 positive cells. The former are the LT-HSC and the latter the ST-HSC/MPP. In these studies, there was no selection for Thy-1.1 and, thus, the ST-HSC population will also include multipotent progenitors (MPP). Both these classes of stem cells are short term repopulators and are designated here as ST-HSC/MPP. The isolated LT-HSC or ST-HSC/MPP were then competed against C57BL/6J marrow into lethally irradiated C57BL/6J host mice. Engraftment and lineage analysis was determined by staining peripheral blood with CD45.1, CD45.2, myeloid markers GR-1, and CD11b or lymphoid markers B220 and CD3 as outlined in the Methods section.

**Figure 2:**
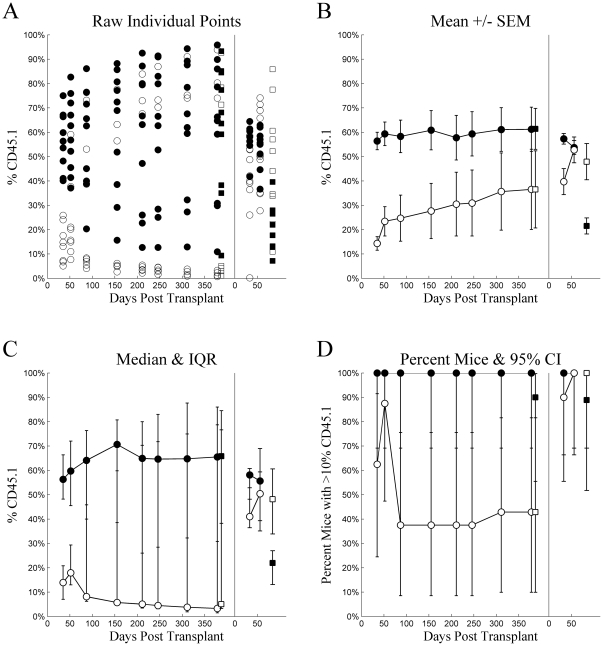
Experiment 1: intravenous injection. Chimerism (%CD45.1) (ordinate) plotted as a function of Days Post Transplant (abscissa), type of cells injected (•▪: LT-HSC, ○□: ST-HSC), and source (•○: whole blood, ▪□: whole bone marrow) for several different methods of summary: A) raw individual mouse percent CD45 cells that were CD45.1, B) mean ± standard error of the mean, C) median with inter-quartile range, and D) percent of mice with greater than 10% of CD45 cells being CD45.1. For each tile, the data from the primary transplant is plotted on the left and secondary transplant on the right, separated by a vertical line at zero for the secondary transplant.

Figure 2 plots several visualizations for chimerism from Experiment 1. Individual mice, (Tile A) in both LT-HSC and ST-HSC, exhibit bimodality over most time points with several mice exhibiting higher chimerism while others show low. While individual mice were not tracked, it is likely this results from engraftment “taking” in some, but not others. This includes 3 mice injected with ST-HSC that exhibited exceptional chimerism, and 3 mice injected with LT-HSC that showed poor or declining chimerism. Consider these observations while examining Tile B. In particular, note that the means for both groups of mice are located in areas where virtually no individual mice observations were located. Indeed, even the area covered when considering standard error of the mean spans values that correspond to few, if any, individual mice values over most time points. It can be argued that the central tendency of the LT-HSC is underestimated and that of the ST-HSC is overestimated, while variability was underestimated for both. These observations demonstrate the inappropriateness of single means and estimates of their variability for describing bimodally distributed data. Two alternatives are presented in Tiles C and D. Tile C indicates central tendency using the median and variability based on the inter-quartile range, which more appropriately spans the bimodal data. These compare much more favorably to the individual observations and most closely correspond to the distribution-free statistics used to compare groups. Tile D dichotomizes mice into successful engraftment vs. failed engraftment by employing a relatively arbitrary, but conservative threshold of 10% chimerism, plotting this proportion for each time point along with its binomial 95% confidence interval. However, with such small sample sizes, this option was considered overly conservative for analysis. Similar 4-tiled figures are presented for each injection method for all experiments. However, Figure 2 most clearly demonstrates the issue of bimodality in the data, likely because its overall levels of chimerism were higher than in other experiments.

Based on the distribution-free Wilcoxon rank-sum statistic (with Holm adjustment for multiple comparisons), LT-HSC had significantly higher chimerism at 35 (adj.p<0.001) and 52 (adj.p = 0.011) days post transplant in the primary recipients compared to later time points (adj.p≥0.05 for all). When whole bone marrow harvested from the primary recipients was pooled and subsequently transplanted into the secondary recipients, LT-HSC had better chimerism than ST-HSC/MPP at Day 33 (adj.p = 0.006), but not at Day 56 (adj.p = 0.842). Interestingly, when whole bone marrow was harvested from secondary recipients at Day 84, ST-HSC/MPP chimerism was significantly *higher* than that of LT-HSC (adj.p = 0.020).

**Figure 3:**
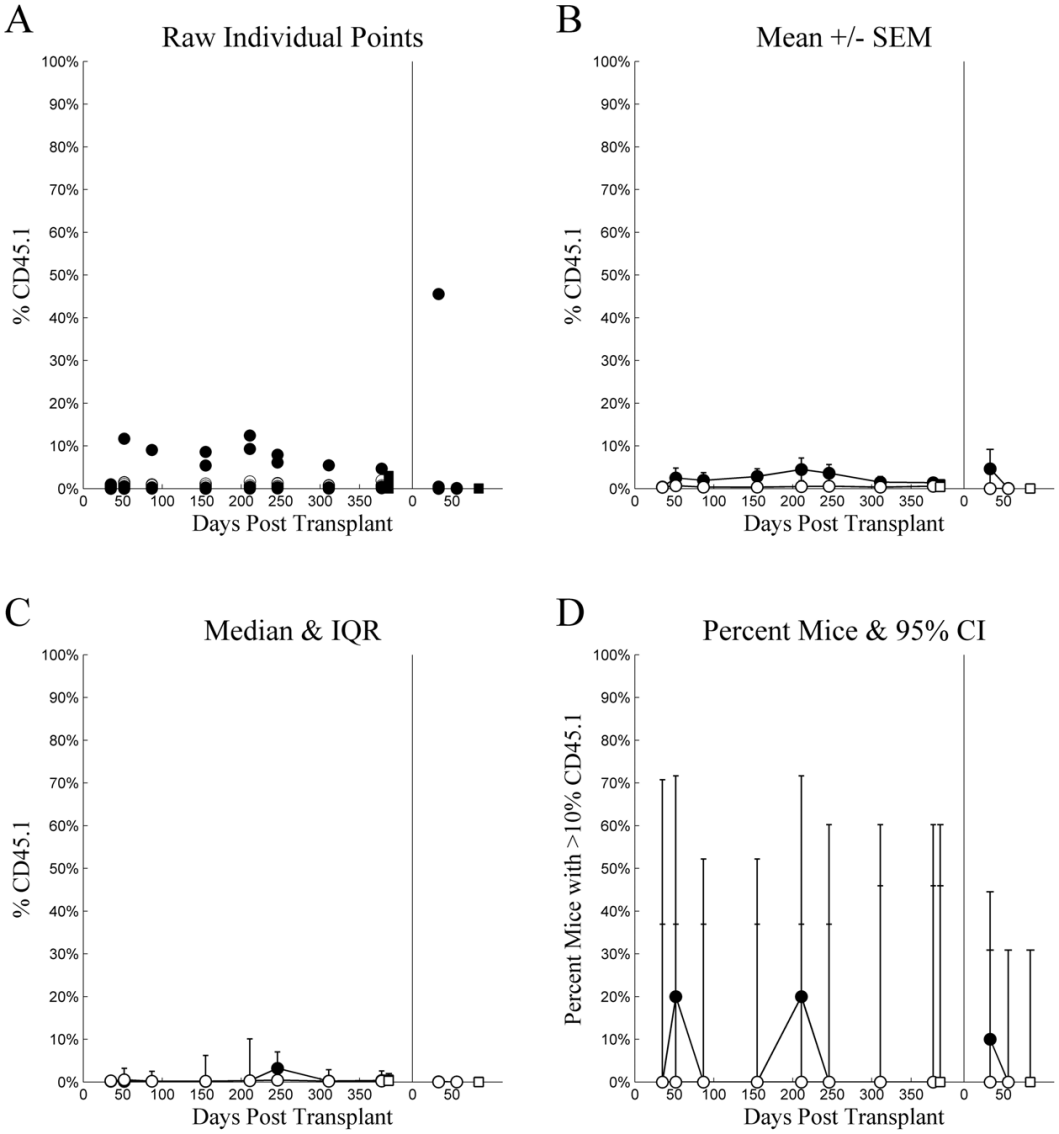
Experiment 1: intraperitoneal injection. Chimerism (%CD45.1) (ordinate) plotted as a function of Days Post Transplant (abscissa), type of cells injected (•▪: LT-HSC, ○□: ST-HSC), and source (•○: whole blood, ▪□: whole bone marrow) for several different methods of summary: A) raw individual mouse percent CD45 cells that were CD45.1, B) mean ± standard error of the mean, C) median with inter-quartile range, and D) percent of mice with greater than 10% of CD45 cells being CD45.1. For each tile, the data from the primary transplant is plotted on the left and secondary transplant on the right, separated by a vertical line at zero for the secondary transplant.

Engraftment was very low in mice receiving intraperitoneal injections. These levels were so low as to defy statistical analyses. Figure 3 is presented to demonstrate the low levels and for the sake of completeness and consistency.

**Figure 4:**
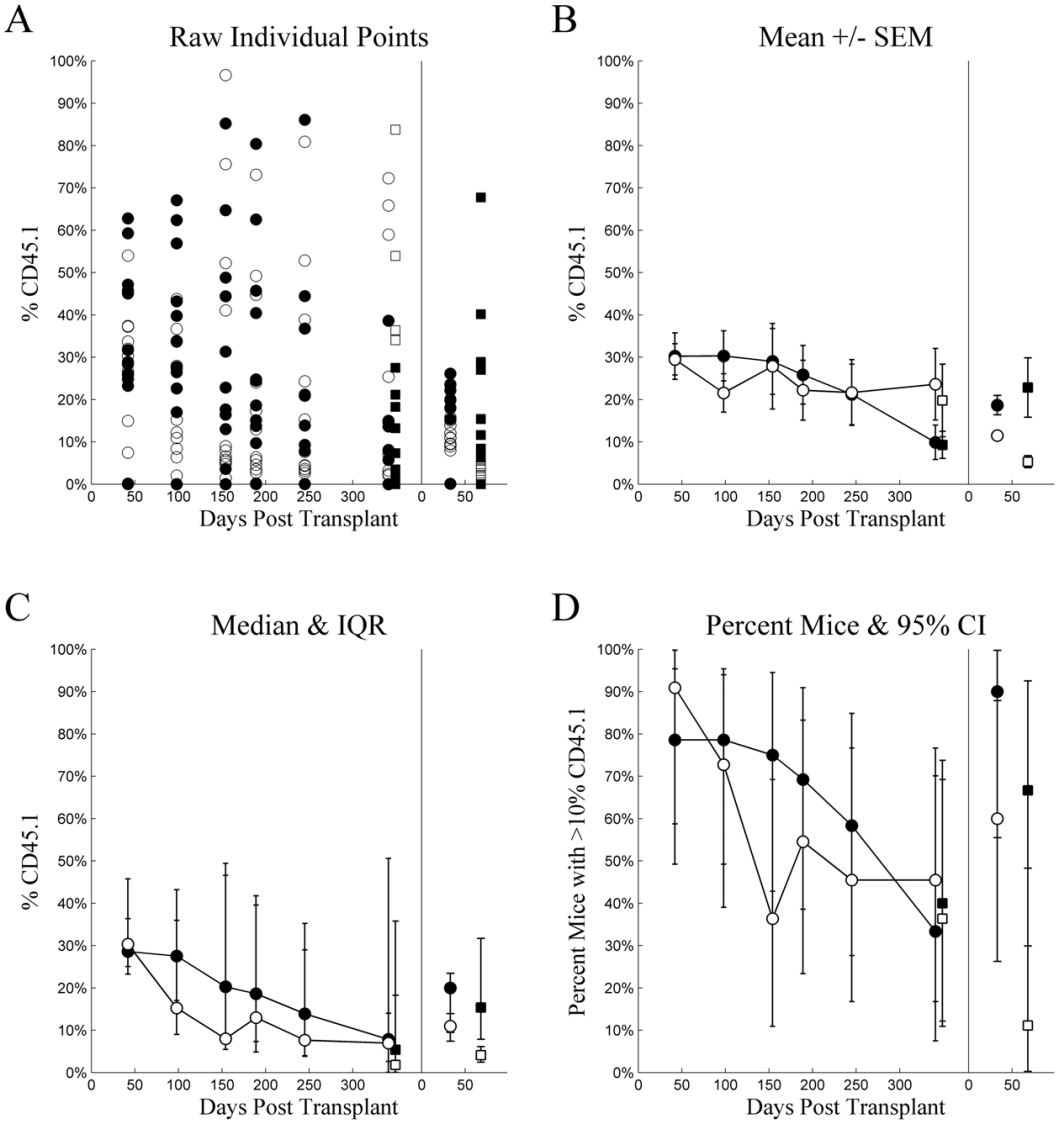
Experiment 2: intravenous injection. Chimerism (%CD45.1) (ordinate) plotted as a function of Days Post Transplant (abscissa), type of cells injected (•▪: LT-HSC, ○□: ST-HSC), and source (•○: whole blood, ▪□: whole bone marrow) for several different methods of summary: A) raw individual mouse percent CD45 cells that were CD45.1, B) mean ± standard error of the mean, C) median with inter-quartile range, and D) percent of mice with greater than 10% of CD45 cells being CD45.1. For each tile, the data from the primary transplant is plotted on the left and secondary transplant on the right, separated by a vertical line at zero for the secondary transplant.

**Figure 5:**
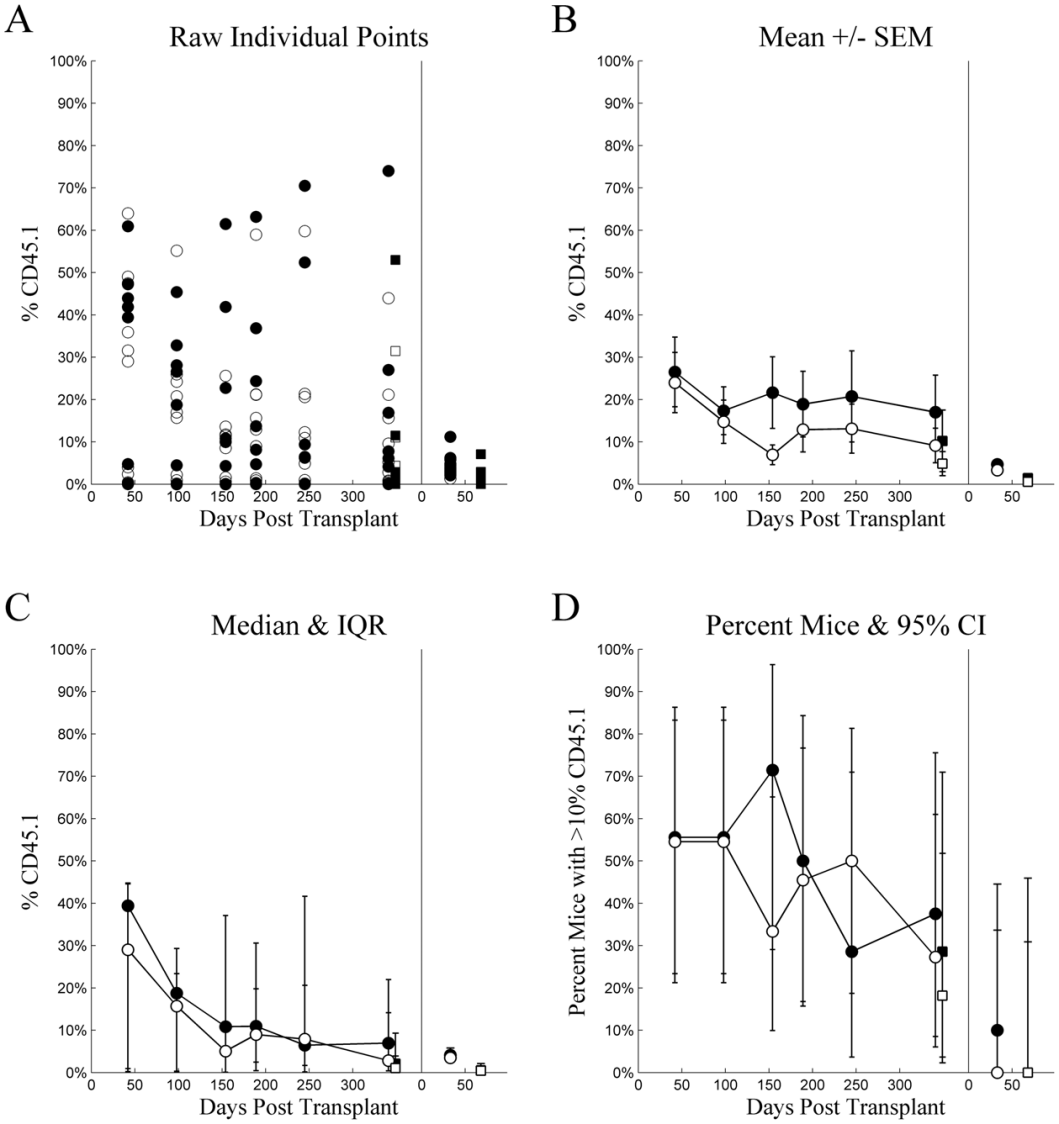
Experiment 2: intrafemoral injection. Chimerism (%CD45.1) (ordinate) plotted as a function of Days Post Transplant (abscissa), type of cells injected (•▪: LT-HSC, ○□: ST-HSC), and source (•○: whole blood, ▪□: whole bone marrow) for several different methods of summary: A) raw individual mouse percent CD45 cells that were CD45.1, B) mean ± standard error of the mean, C) median with inter-quartile range, and D) percent of mice with greater than 10% of CD45 cells being CD45.1. For each tile, the data from the primary transplant is plotted on the left and secondary transplant on the right, separated by a vertical line at zero for the secondary transplant.

Figures 4 and 5 plot the 4-tiled figures for Experiment 2, intravenous and intrafemoral injections, respectively. Statistical analyses made comparisons between the 4 groups defined by cell type (LT-HSC vs. ST-HSC/MPP) and injection method. There were no statistically significant differences between groups at any time point in primary recipients (adj.p≥0.05 for all). Again, the wide inter-quartile ranges more appropriately span the bimodal data. In the secondary recipients, however, LT-HSC injected intravenously had higher chimerism than any other group at 33 days (adj.p<0.05 for all), and ST-HSC injected intravenously had higher chimerism than the cell types injected intrafemorally (adj.p<0.05 for both). At Day 68, LT-HSC and ST-HSC/MPP did not differ significantly for either injection method (adj.p≥0.05 for both). LT-HSC injected intravenously had higher chimerism than LT-HSC injected intrafemorally (adj.p = 0.028). In contrast, ST-HSC/MPP injected intravenously had significantly higher chimerism than either LT-HSC or ST-HSC/MPP injected intrafemorally (adj.p<0.05 for both). The failure of LT-HSC intravenously injected to reach statistical significance as compared to ST-HSC/MPP injected intrafemorally, despite having a higher median than ST-HSC/MPP injected intravenously, reflected the wider variation in chimerism for LT-HSC than ST-HSC/MPP, as suggested by the wider inter-quartile range. This comparison was actually statistically significant prior to adjustment for multiple comparisons with the Holm Test (unadjusted p = 0.0133).

The above two experiments involved “single sorts”, but in order to further purify our marrow cells, in this third experiment we performed a “double sort”.

**Figure 6:**
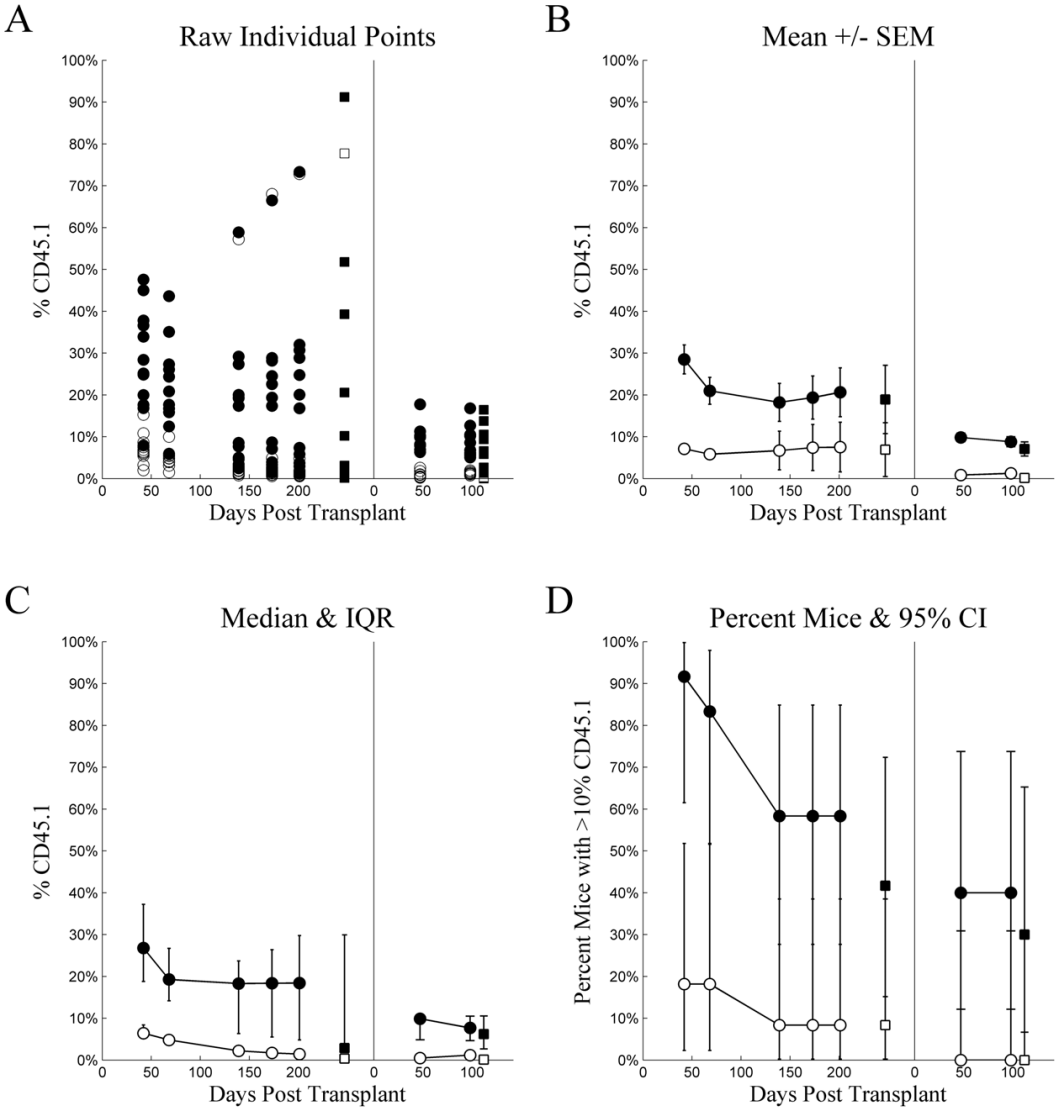
Experiment 3: intravenous injection. Chimerism (%CD45.1) (ordinate) plotted as a function of Days Post Transplant (abscissa), type of cells injected (•▪: LT-HSC, ○□: ST-HSC), and source (•○: whole blood, ▪□: whole bone marrow) for several different methods of summary: A) raw individual mouse percent CD45 cells that were CD45.1, B) mean ± standard error of the mean, C) median with inter-quartile range, and D) percent of mice with greater than 10% of CD45 cells being CD45.1. For each tile, the data from the primary transplant is plotted on the left and secondary transplant on the right, separated by a vertical line at zero for the secondary transplant.

**Figure 7:**
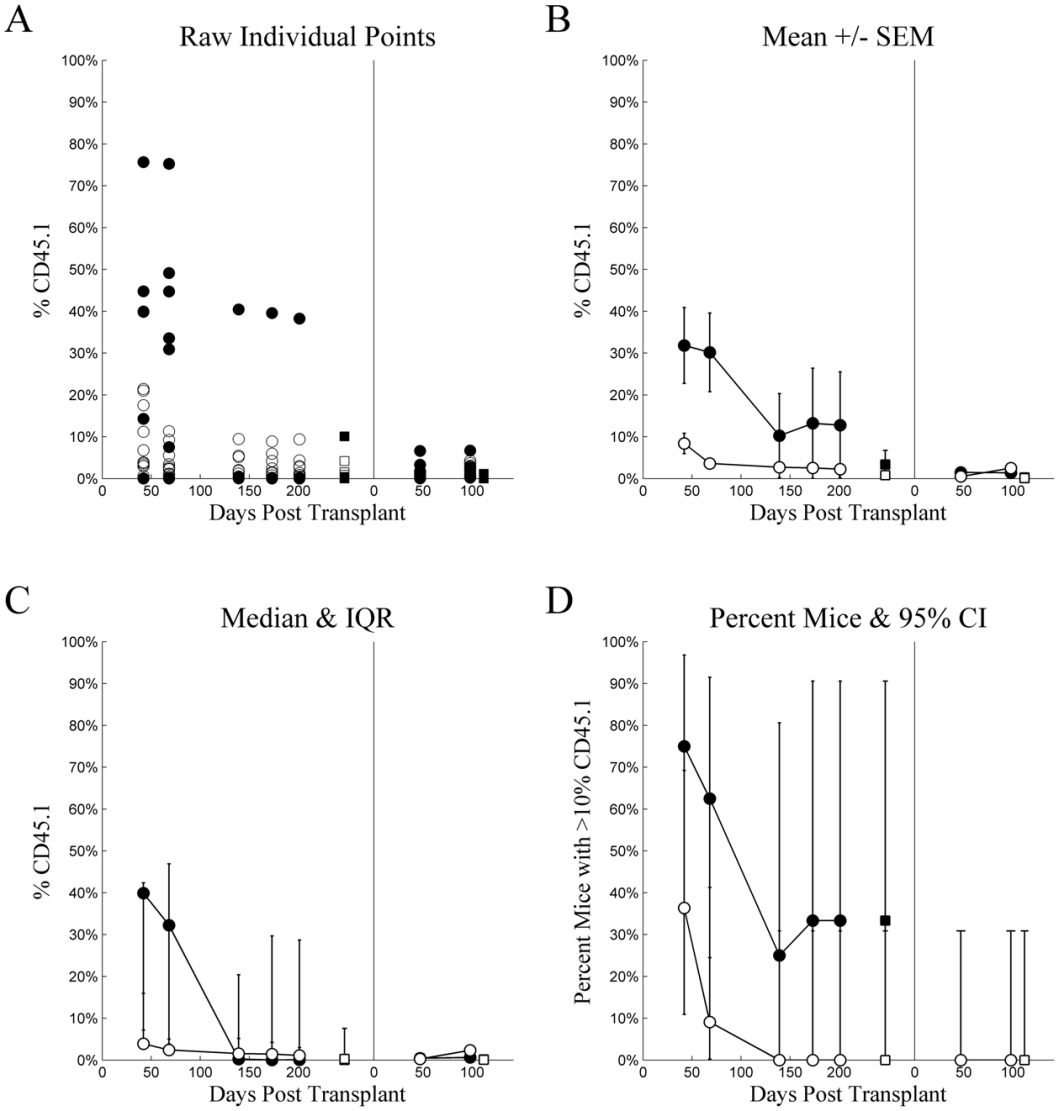
Experiment 3: intrafemoral injection. Chimerism (%CD45.1) (ordinate) plotted as a function of Days Post Transplant (abscissa), type of cells injected (•▪: LT-HSC, ○□: ST-HSC), and source (•○: whole blood, ▪□: whole bone marrow) for several different methods of summary: A) raw individual mouse percent CD45 cells that were CD45.1, B) mean ± standard error of the mean, C) median with inter-quartile range, and D) percent of mice with greater than 10% of CD45 cells being CD45.1. For each tile, the data from the primary transplant is plotted on the left and secondary transplant on the right, separated by a vertical line at zero for the secondary transplant.

Figures 6 and 7 plot the 4-tiled figures for Experiment 3, intravenous and intrafemoral injections, respectively. In contrast to Experiment 2, there were statistically significant differences between groups at all points in time for both primary and secondary recipients. These were consistent and represented the differences between LT-HSC injected intravenously and ST-HSC/MPP injected via either method in primary recipients. All such comparisons were statistically significant (adj.p<0.05) excepting the comparisons with ST-HSC/MPP injected intravenously at Days 173 and 201 (adj.p = 0.057 and adj.p = 0.068, respectively), which were not statistically significant after adjustment with the Holm Test (unadjusted p = 0.002 and p = 0.003, respectively). No other comparisons were statistically significant within primary recipients. The pattern of superiority in intravenously injected LT-HSC continued and developed further in secondary recipients. Here, LT-HSC intravenously injected had higher chimerism at all three points in time (adj.p<0.05 for all), with the only additionally statistically significant difference being between short-term intravenously and intrafemorally injected cells at Day 98 (adj.p = 0.0369).

**Figure 8:**
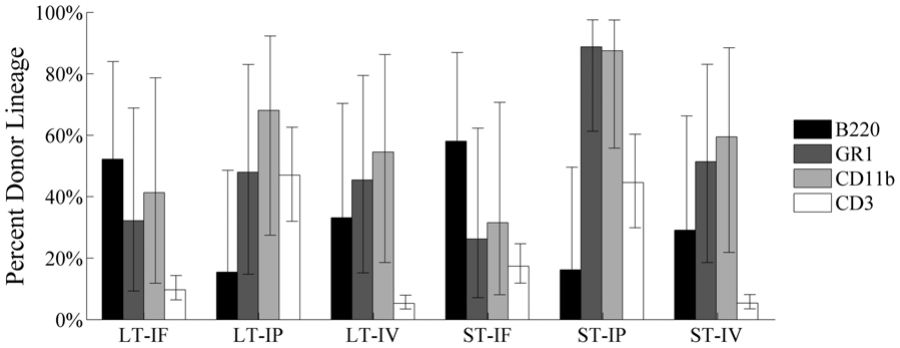
Multilineage nature of engraftment: Summary graph of all primary transplant experiments showing that when engraftment occurred, multiple cell types were seen. Therefore, points displayed represent percent of cells from donor. Serial transplants of the primary whole bone marrow were also multilineage in nature when engraftment occurred (data not shown).

In all three experiments, engraftment for LT-HSC and ST-HSC/MPP was observed to be multilineage in nature as shown in Figure 8 and described in Methods. For each experiment, peripheral blood was analyzed for percent donor contribution to myeloid lineages, represented by CD11b^+^ and GR1^+^ cells, and lymphoid lineages, represented by B220^+^ and CD3^+^ cells, at various time points. For Experiments 1 and 2, analyses were done at 2, 4, 6, 8, and 12 months post-transplant. For Experiment 3, analysis was done at 1, 2, 4, 6, and 8 months following transplant. At all time points for all experiments, when donor engraftment occurred, this engraftment was multilineage. The donor contribution to myeloid and lymphoid lineage was stable over time in both primary and secondary transplants and similar to results seen with whole bone marrow analysis (data not shown).

## Discussion

Our studies indicate that changes in the route of administration of ST-HSC/MPP to intraperitoneal or intrafemoral routes, in general, did not result in engraftment equivalent to that seen when LT-HSC were administered intravenously, thus indicating that differences between engraftment of these cell types was not due to differences in homing efficiency. However, this point is essentially moot since the ST-HSC/MPP cells showed prolonged multilineage engraftment and secondary engraftment. This is in contrast to previous reports of short-term engraftment of marrow cells selected based on lineage negativity, and c-kit, Sca-1, and Flk-2 positivity. The expression of Flk-2 has correlated with stem cell differentiation and loss of self renewal capacity and generally marks the transition from LT-HSC to ST-HSC/MPP, although some cells with characteristics of LT-HSC may express low levels of Flt3 [14]. Cells isolated, as described here in the standard lineage, would be considered to be at the ST-HSC or MPP levels and not have the capacity for long-term multilineage repopulation.

There have been a number of studies analyzing various characteristics of ST-HSC/MPP but, in general, these studies have not included confirmation of the *in vivo* engraftment phenotype, but rather utilized the cell surface phenotype and then studied cells separated on this basis.

In separate studies, we have forwarded a non-hierarchical model of stem/progenitor cell regulation based on observations of a continuously and reversibly changing cell phenotype tied to cell cycle status [23]–[36]. In this model, phenotype lability of stem cells is assumed. We have documented reversible changes of stem cells as to engraftment [1], [24], [25], [27], differentiation [34], marrow homing [30], adhesion protein and cytokine receptor expression [28], [29], [31], and gene expression linked to cell cycle phase [1], [23], and our recent work has also indicated that the long-term repopulating cell in the marrow is a cycling cell. (Unpublished observations: Quesenberry PJ, Dooner MS, Goldberg LR, 2009.) Earlier work on colony-forming unit-spleen (CFU-s) by Necas and Znojil [37] indicated that this putative stem cell showed marked quantitative changes and alterations in cell cycle status in different experiments and between individual mice. These investigators proposed that there might be bursts of cycle activity separate from circadian changes. Lastly, we have previously documented marked changes in 10 week engraftment at different circadian times, and this also changed on a seasonal basis [38]. Thus, there are many potential explanations for these results not matching those initially reported, all suggesting that there are many important variables which have not been considered in a number of previous experiments.

Recent work has emphasized the importance of CD150, CD48, or CD41 in stem cell separations, and these were not utilized in our current set of experiments [39], [40]. However, this is appropriate, because these also were not utilized in the initial experiments defining LT-HSC and ST-HSC/MPP. In addition, the utilization of another surface marker to subset stem cells, simply, is a further measure of the heterogeneity of the stem cell population and does not provide a truer picture of the stem cell [41]. As we have proposed earlier, stem cell purifications leave behind most stem cells and this is not a random procedure [42]. Thus, it is questionable whether a marrow cell purified on the basis of surface epitope selection is truly representative of marrow stem cells in the whole organism. Our work on determining the cell cycle status of purified and whole marrow cells indicates that quite different cycle results are obtained with the cell cycle status of purified as opposed to unseparated marrow stem cells. It is of interest that there remains some controversy as to the ultimate meaning of CD150 marking for stem cells: we feel that the addition of this marker adds little to our understanding of hematopoietic marrow stem cells. Finally, a very intriguing report suggested that antibody binding to stem cells in these separations might have very significant inhibitory effects on their engraftment capacity [43]. These studies imply that antibody based purification of stem cells is inherently flawed, however, a major question in these studies is whether the antibody binding altered the stem cell separation and, thus, these results at present are not definitive. Follow-up studies can resolve this question. Finally, it is possible that methodologic differences between studies could underlie the different results. The standard LT-HSC/ST-HSC separations, as described by the Weissman group, did not employ an initial preparative density separation which was utilized in the current study. Overall, it is fair to state that there are many unanswered questions as to the meaning and validity of stem cell separations.

In summary, we did not find that homing differences explained differences in engraftment capacity between marrow cells differing in Flk-2 expression, and we have found that the classic ST-HSC/MPP phenotype in our experiments does not match that initially reported. More generally, we would expect to see a great deal of stem cell phenotype variation depending upon cell cycle status, circadian time, and other stochastic influences. We feel that the previously defined hierarchical stem cell classes actually represent a very fluid population, and that surface phenotype does not accurately define stem cell populations. We propose that a major challenge to the field is not to purify stem cells but rather to define the whole stem cell population.

## Materials and Methods

### Mice

Six- to eight-week-old, congenic, male B6.SJL-*Ptprc^a^Pepc^b^*/BoyJ (B6.SJL) and male C57BL/6J mice were purchased from The Jackson Laboratory (Bar Harbor, ME). Animals were housed for at least one week prior to use in a conventional clean facility with 12 hour light-dark cycles and access to mouse chow and water ad libitum in accordance with the National Research Council's *Guide for the Care and Use of Laboratory Animals* [44]. All procedures were approved by the Rhode Island Hospital Institutional Animal Care and Use Committee.

### LT-HSC and ST-HSC/MPP Purification

B6.SJL mice, 10–12 weeks of age, anesthetized with isoflurane, were killed by cervical dislocation. Bone marrow was harvested from the iliac crests, femurs, tibiae, and spines by crushing in a mortar with a pestle and 1× phosphate buffered saline containing 5% heat-inactivated fetal calf serum and 1% penicillin-streptomycin (PBS-HIFCS), as previously described [45]. The crushed marrow was washed extensively with fresh PBS-HIFCS. The wash volumes held in 50 ml conical tubes were filtered through 40 µm nylon cell strainers to remove small bone particles and were pooled for a cell count and centrifugation. The cell pellet was resuspended in fresh PBS-HIFCS to a final concentration of 1×10^7^ cells per ml. Each 30 ml of cell suspension was mixed with 10.1 ml of Optiprep™ (AXIS-SHIELD PoC AS, Oslo, Norway) density gradient solution and 4 ml of sterile, distilled water in 50 ml conical tubes. Each tube was overlaid with 5 ml PBS-HIFCS and centrifuged at 1000× g for 30 minutes without acceleration or braking. A low-density leukocyte cell fraction (<1.077 g/ml) was isolated per manufacturer's instructions. This fraction was washed and then lineage depleted by incubation with rat IgG anti-mouse monoclonal antibodies to B220, CD4, CD8a, CD11b, TER119 [0.1 mg/10^6^ cells], CD3, CD5 [0.25 mg/10^6^ cells], and GR1 [0.5 mg/10^6^ cells] (BD Pharmingen, San Diego, CA except CD3 which is from SouthernBiotech, Birmingham, AL). The lineage positive cells were removed with Dynabeads®, sheep anti-rat IgG-conjugated super-paramagnetic polystyrene beads (4.5 µm in diameter) (Dynal® Magnetic Bead System, Invitrogen, Carlsbad, CA) per manufacturer's instructions. Immunomagnetic bead-rosetted cells were removed using a magnetic particle concentrator (Dynal®, MPC-6), and the unrosetted cells remaining in suspension were harvested by pipette. These were the lineage negative cells. They were transferred to a fresh tube and incubated with: biotin-conjugated Sca-1, allophycocyanin (APC)-conjugated c-kit, phycoerythrin (PE)-conjugated Flk-2 (BD Pharmingen, San Diego, CA), and FITC-conjugated rat polyclonal IgG (eBioscience, San Diego, CA) [0.25 µg/10^6^ cells]. The cells were washed with PBS-HIFCS and incubated with streptavidin-Alexa Fluor 405 [0.5 µg/10^6^ cells] (Invitrogen, Carlsbad, CA) for 15 minutes on ice. Following a final wash in PBS-HIFCS, the cells were re-suspended to a concentration of 50×10^6^ cells/ml in a 1 µg/ml propidium iodide solution (PI) (Sigma, St. Louis, MO).

### Fluorescence-Activated Cell Sorting (FACS)

LT-HSC were isolated by flow cytometry and defined as follows: lineage negative (FITC negative), c-kit (APC) positive, Sca-1 (Alexa Fluor 405) positive, and Flk-2 (PE) negative. ST-HSC/MPP were defined as lineage negative (FITC negative), c-kit (APC) positive, Sca-1 (Alexa Fluor 405) positive, and Flk-2 (PE) positive. Dead cells were gated out based on high PI staining. Cell sorting was performed using a BD Influx™ cell sorter (BD Bioscience, San Diego, CA) equipped with the following: a 100 mW 488 nm sapphire, a 100 mW UV, a 405 nm violet, a 635 nm red, and a 561 nm green/yellow lasers. Propidium iodide was excited using the UV laser and emission collected through a 670/30 nm filter. The 488 nm laser was used to excite FITC, while PE was excited by the 561 nm laser. Fluorescent emission was collected using 528/38 nm and 610/20 nm filters, respectively. Alexa Fluor 405 was excited using the 405 nm laser, and emission was collected through a 460/50 nm filter. The 635 nm red laser was used to excite APC, and the fluorescent emission was collected through a 670/30 nm filter.

The cell suspension was gently vortexed and kept at 4°C throughout the sort. A gate was set around cells in the side scatter versus forward scatter plot (Figure 1A). Dying or dead cells were gated out based on high PI staining (Figure 1B). Any remaining FITC-tagged lineage positive/low cells were then gated out (Figure 1C). Sca-1/c-kit double positive cells were gated in (Figure 1D). These cells were then sorted into separate tubes based on Flk-2 staining (Figure 1E). In one experiment, the Flk-2 negative and the Flk-2 positive sorted cell populations were resorted individually to confirm purity based on their Flk-2 staining (Figure 1 F1,F2 and G1,G2) respectively.

### Irradiation and Stem Cell Transplantation

C57BL/6J recipient mice received 950 cGy irradiation (Gammacell® 40 Exactor, ^137^Cesium source irradiator) (Best Theratronics Ltd., Ottawa, Ontario, Canada) in two fractions, three hours apart, at a rate of 107 cGy per minute. Cells were infused within three hours post irradiation. Each mouse received either 200–500 LT-HSC (B6.SJL) combined with 300,000 C57BL/6J competitor whole marrow cells or 200–500 ST-HSC/MPP (B6.SJL) combined with 300,000 C57BL/6J competitor cells by one of three injection routes: intravenous (IV), intraperitoneal (IP), or intrafemoral (IF). The IV and IP volumes were 0.5 ml per animal, and the IF volume was 0.02 ml per anesthetized animal. Prior to IF injection, mice were anesthetized using ketamine (75 mg/kg) and medetomidine (1 mg/kg) intraperitoneally. Once anesthetized, the region from the inguen to the knee was sterilized with 70% ethyl alcohol and 10% povidone iodine (Betadine). With one hand, the knee was flexed to 90°, effectively securing the leg to prevent motion while performing the procedure. With the other hand, a 27-gauge U-100 needle (Terumo Medical Corporation, Elkton, MD) was carefully inserted into the joint surface of the distal femur above the patella, through the patellar tendon, and then the stem cells were slowly injected into the bone marrow cavity [46]–[48].

### Serial Secondary Whole Bone Marrow Transplantation

At eight months or one year after cell infusion, the mice from the primary transplant experiments were sacrificed and whole bone marrow from femurs and tibiae was harvested. The marrow from each group was pooled, and 6×10^6^ cells per mouse were serially transplanted via tail vein injection to a new set of lethally irradiated mice. Engraftment of B6.SJL cells was analyzed at multiple time points after secondary transplant (vide infra).

### Engraftment and Lineage Analysis

Peripheral blood from primary or secondarily engrafted mice was obtained at varying times from the tail vein, utilizing EDTA as an anticoagulant. Chimerism and the lineage of the repopulating cells were determined by flow cytometric analysis. Whole blood was split into two aliquots and labeled for 30 minutes with either, CD45.1 FITC, CD45.2 PerCP-Cy5.5, B220 PE, and CD3 APC antibodies (lymphoid lineage master mix) or CD45.1 FITC, CD45.2 PerCP-Cy5.5, CD11b PE, and GR-1 APC antibodies (myeloid lineage master mix) (BD Pharmingen, San Diego, CA) in PBS pH 7.2 containing 0.5% FBS and 0.2% sodium azide (FACS buffer). The samples were subsequently incubated with 1× Pharm Lyse™ ammonium chloride-based lysing reagent (BD Pharmingen, San Diego, CA) and washed with FACS buffer to remove red blood cells. Samples were resuspended in FACS buffer and analyzed on a BD LSR II™ flow cytometer (BD Bioscience, San Jose, CA). A plot of forward and side scatter was gated to eliminate debris and a plot of forward scatter area and width to eliminate doublets. A minimum of 10,000 events were collected. Appropriate compensation controls were set to avoid spectral overlap. The percentage of CD45.1 (donor) engrafted was determined by dividing the number of CD45.1 positive events by the sum of CD45.1 positive plus CD45.2 positive events and multiplying this result by 100.

In order to determine the lineage of the transplanted repopulating cells, all CD45.1 positive cells were analyzed for expression of the lymphoid markers B220 and CD3 and for the expression of the myeloid markers CD11b and GR-1. For example, the percentage of B220 positive donor cells was determined by dividing the number of CD45.1 positive, B220 positive cells by the sum of CD45.1 positive, B220 positive cells and CD45.1 positive, B220 negative cells and multiplying this value by 100.

### Statistical Methods

The distribution of percent chimerism was not normal and frequently bimodal, with a portion of the group exhibiting one pattern (e.g., higher) than the rest of the group. This was particularly apparent in the first experiment, where chimerism was overall higher. The bimodal nature of the data made the use of standard parametric statistics inappropriate (e.g., t-test or analysis of variance). Therefore, distribution-free statistics based on rank-sums were used. Unfortunately, there are currently no non-parametric equivalents to the parametric statistics capable of analyzing higher order designs, particularly not ones with repeated measures (e.g., two-way analysis of variance or mixed models). Therefore, independent rank-sum statistics were used to compare groups at each time point, with p-values adjusted for alpha inflation using the Holm test [49], as described in more detail below.

There were only 2 groups of animals in Experiment 1 because intraperitoneal injection data was not analyzed. The analysis of this experiment used a single Exact Wilcoxon Rank-Sum test at each time point. Experiments 2 and 3 included 4 groups of animals. Here, the Kruskal-Wallis test was used as an omnibus test for any differences between groups. If this was statistically significant, then follow-up pair-wise comparisons were made between the 4 groups using the Exact Wilcoxon Rank-Sum test. The p-values of all comparisons within an experiment were adjusted for Type I error inflation using the Holm test. The relatively large number of comparisons makes this technique somewhat conservative and, so, unadjusted p-values are noted occasionally in addition to the adjusted p-values when results may seem counterintuitive or otherwise inconsistent, with possible Type II error implicated.

The occurrence of bimodality presented a difficult analytic problem, which was mirrored in the choice as to how to visualize the results. Presentation of the mean of bimodal data is particularly problematic. Therefore, a 4-tiled figure was created for each experiment and injection protocol in which several methods for visualizing are contrasted, including the conventional mean-based technique. The four tiles present visualizations: A) the raw individual points for each mouse, B) the mean ± standard error of the mean, C) the median with inter-quartile range, and D) the percent of mice exceeding 10% chimerism with 95% confidence intervals. Each is plotted as a function of days post transplant, cell type transplanted (LT-HSC vs. ST-HSC), and source (whole blood vs. whole bone marrow). The third tile, labeled “C”, was chosen as the closest representation to the statistical analyses conducted.

## References

[1] Lambert JF, Liu M, Colvin GA, Dooner M, McAuliffe CI (2003). Marrow stem cells shift gene expression and engraftment phenotype with cell cycle transit.. J Exp Med.

[2] Forsberg EC, Prohaska SS, Katzman S, Heffner GC, Stuart JM (2005). Differential expression of novel potential regulators in hematopoietic stem cells.. PLoS Genet.

[3] Rossi DJ, Bryder D, Zahn JM, Ahlenius H, Sonu R (2005). Cell intrinsic alterations underlie hematopoietic stem cell aging.. Proc Natl Acad Sci USA.

[4] Arber C, BitMansour A, Sparer TE, Higgins JP, Mocarski ES (2003). Common lymphoid progenitors rapidly engraft and protect against lethal murine cytomegalovirus infection after hematopoietic stem cell transplantation.. Blood.

[5] Manz MG, Miyamoto T, Akashi K, Weissman IL (2002). Prospective isolation of human clonogenic common myeloid progenitors.. Proc Natl Acad Sci USA.

[6] Miyamoto T, Iwasaki H, Reizis B, Ye M, Graf T (2002). Myeloid or lymphoid promiscuity as a critical step in hematopoietic lineage commitment.. Dev Cell.

[7] Christensen JL, Weissman IL (2001). Flk-2 is a marker in hematopoietic stem cell differentiation: A simple method to isolate long-term stem cells.. Proc Natl Acad Sci USA.

[8] Kondo M, Scherer DC, King AG, Manz MG, Weissman IL (2001). Lymphocyte development from hematopoietic stem cells.. Curr Opin Genet Dev.

[9] Kondo M, Scherer DC, Miyamoto T, King AG, Akashi K (2000). Cell-fate conversion of lymphoid-committed progenitors by instructive actions of cytokines.. Nature.

[10] Akashi K, Traver D, Miyamoto T, Weissman IL (2000). A clonogenic common myeloid progenitor that gives rise to all myeloid lineages.. Nature.

[11] Cheshier SH, Morrison SJ, Liao X, Weissman IL (1999). *In vivo* proliferation and cell cycle kinetics of long-term self-renewing hematopoietic stem cells.. Proc Natl Acad Sci USA.

[12] Kondo M, Weissman IL, Akashi K (1997). Identification of clonogenic common lymphoid progenitors in mouse bone marrow.. Cell.

[13] Morrison SJ, Wandycz AM, Hemmati HD, Wright DE, Weissman IL (1997). Identification of a lineage of multipotent hematopoietic progenitors.. Development.

[14] Adolfsson J, Mansson R, Buza-Vidas N, Hultquist A, Liuba K (2005). Identification of Flt3^+^ lympho-myeloid stem cells lacking erythro-megakaryocytic potential: A revised road map for adult blood lineage commitment.. Cell.

[15] Randall TD, Lund FE, Howard MC, Weissman IL (1996). Expression of murine CD38 defines a population of long-term reconstituting hematopoietic stem cells.. Blood.

[16] Morrison SJ, Weissman IL (1994). The long-term repopulating subset of hematopoietic stem cells is deterministic and isolatable by phenotype.. Immunity.

[17] Forsberg EC, Bhattacharya D, Weissman IL (2006). Hematopoietic stem cells: Expression profiling and beyond.. Stem Cell Rev.

[18] Warren L, Bryder D, Weissman IL, Quake SR (2006). Transcription factor profiling in individual hematopoietic progenitors by digital RT-PCR.. Proc Natl Acad Sci USA.

[19] Bryder D, Rossi DJ, Weissman IL (2006). Hematopoietic stem cells: The paradigmatic tissue-specific stem cell.. Am J Pathol.

[20] Forsberg EC, Serwold T, Kogan S, Weissman IL, Passegue E (2006). New evidence supporting megakaryocyte-erythrocyte potential of Flk2/Flt3^+^ multipotent hematopoietic progenitors.. Cell.

[21] Passegue E, Wagers AJ, Giuriato S, Anderson WC, Weissman IL (2005). Global analysis of proliferation and cell cycle gene expression in the regulation of hematopoietic stem and progenitor cell fates.. J Exp Med.

[22] Wagers AJ, Weissman IL (2006). Differential expression of α2 Integrin separates long-term and short-term reconstituting Lin^−/lo^Thy1.1^lo^c-kit^+^Sca-1^+^ hematopoietic stem cells.. Stem Cells.

[23] Dooner GJ, Colvin GA, Dooner MS, Johnson KW, Quesenberry PJ (2008). Gene expression fluctuations in murine hematopoietic stem cells with cell cycle progression.. J Cell Physiol.

[24] Peters SO, Kittler EL, Ramshaw HS, Quesenberry PJ (1995). Murine marrow cells expanded in culture with IL-3, IL-6, IL-11, and SCF acquire an engraftment defect in normal hosts.. Exp Hematol.

[25] Peters SO, Kittler EL, Ramshaw HS, Quesenberry PJ (1996). Ex vivo expansion of murine marrow cells with Interleukin-3 (IL-3), IL-6, IL-11, and stem cell factor leads to impaired engraftment in irradiated hosts.. Blood.

[26] Kittler EL, Peters SO, Crittenden RB, Debatis ME, Ramshaw HS (1997). Cytokine-facilitated transduction leads to low-level engraftment in nonablated hosts.. Blood.

[27] Habibian HK, Peters SO, Hsieh CC, Wuu J, Vergilis K (1998). The fluctuating phenotype of the lymphohematopoietic stem cell with cell cycle transit.. J Exp Med.

[28] Becker PS, Nilsson SK, Li Z, Berrios VM, Dooner MS (1999). Adhesion receptor expression by hematopoietic cell lines and murine progenitors: Modulation by cytokines and cell cycle status.. Exp Hematol.

[29] Berrios VM, Dooner GJ, Nowakowski G, Frimberger A, Valinski H (2001). The molecular basis for the cytokine-induced defect in homing and engraftment of hematopoietic stem cells.. Exp Hematol.

[30] Cerny J, Dooner M, McAuliffe C, Habibian H, Stencil K (2002). Homing of purified murine lymphohematopoietic stem cells: A cytokine-induced defect.. J Hematother Stem Cell Res.

[31] Reddy GP, McAuliffe CI, Pang L, Quesenberry PJ, Bertoncello I (2002). Cytokine receptor repertoire and cytokine responsiveness of Ho^dull^/Rh^dull^ stem cells with differing potentials for G_1_/S phase progression.. Exp Hematol.

[32] Colvin GA, Lambert JF, Carlson JE, McAuliffe CI, Abedi M (2002). Rhythmicity of engraftment and altered cell cycle kinetics of cytokine-cultured murine marrow in simulated microgravity compared with static cultures.. In Vitro Cell Dev Biol Anim.

[33] Colvin GA, Lambert JF, Moore BE, Carlson JE, Dooner MS (2004). Intrinsic hematopoietic stem cell/progenitor plasticity: Inversions.. J Cell Physiol.

[34] Colvin GA, Dooner MS, Dooner GJ, Sanchez-Guijo FM, Demers D (2007). Stem cell continuum: Directed differentiation hotspots.. Exp Hematol.

[35] Reddy GP, Tiarks CY, Pang L, Wuu J, Hsieh CC (1997). Cell cycle analysis and synchronization of pluripotent hematopoietic progenitor stem cells.. Blood.

[36] Pang L, Reddy PV, McAuliffe CI, Colvin GA, Quesenberry PJ (2003). Studies on BrdU labeling of hematopoietic cells: Stem cells and cell lines.. J Cell Physiol.

[37] Necas E, Znojil V (1987). CFU-S content and cycling rate in several strains of mice.. Exp Hematol.

[38] D'Hondt L, McAuliffe C, Damon J, Reilly J, Carlson J (2004). Circadian variations of bone marrow engraftability.. J Cell Physiol.

[39] Kent DG, Copley MR, Benz C, Wöhrer S, Dykstra BJ (2009). Prospective isolation and molecular characterization of hematopoietic stem cells with durable self-renewal potential.. Blood.

[40] Weksberg DC, Chambers SM, Boles NC, Goodell MA (2008). CD150^−^ side population cells represent a functionally distinct population of long-term hematopoietic stem cells.. Blood.

[41] Colvin GA, Berz D, Liu L, Dooner MS, Dooner G (2010). Heterogeneity of non-cycling and cycling synchronized murine hematopoietic stem/progenitor cells.. J Cell Physiol.

[42] Quesenberry PJ, Dooner GJ, Dooner MS (2009). Problems in the Promised Land: status of adult marrow stem cell biology.. Exp Hematol.

[43] Gilner JB, Walton WG, Gush K, Kirby SL (2007). Antibodies to stem cell marker antigens reduce engraftment of hematopoietic stem cells.. Stem Cells.

[44] National Research Council (1996). Guide for the care and use of laboratory animals.

[45] Colvin GA, Lambert JF, Abedi M, Hsieh CC, Carlson JE (2004). Murine marrow cellularity and the concept of stem cell competition: geographic and quantitative determinants in stem cell biology.. Leukemia.

[46] Kushida T, Inaba M, Hisha H, Ichioka N, Esumi T (2001). Intra-bone marrow injection of allogeneic bone marrow cells: a powerful new strategy for treatment of intractable autoimmune diseases in MRL/lpr mice.. Blood.

[47] Mazurier F, Doedens M, Gan OI, Dick JE (2003). Rapid myeloerythroid repopulation after intrafemoral transplantation of NOD-SCID mice reveals a new class of human stem cells.. Nat Med.

[48] McKenzie JL, Gan OI, Doedens M, Dick JE (2005). Human short-term repopulating stem cells are efficiently detected following intrafemoral transplantation into NOD/SCID recipients depleted of CD122^+^ cells.. Blood.

[49] Holm S (1979). A simple sequentially rejective multiple test procedure.. Scand J Statist.

